# Simple observation of *Streptococcus mutans* biofilm by scanning electron microscopy using ionic liquids

**DOI:** 10.1186/s13568-015-0097-4

**Published:** 2015-01-24

**Authors:** Yoko Asahi, Jiro Miura, Tetsuya Tsuda, Susumu Kuwabata, Katsuhiko Tsunashima, Yuichiro Noiri, Takao Sakata, Shigeyuki Ebisu, Mikako Hayashi

**Affiliations:** Department of Restorative Dentistry and Endodontology, Osaka University Graduate School of Dentistry, 1-8 Yamadaoka, Suita, Osaka 565-0871 Japan; Division for Interdisciplinary Dentistry, Osaka University Graduate School of Dentistry, 1-8 Yamadaoka, Suita, Osaka 565-0871 Japan; Department of Applied Chemistry, Graduate School of Engineering, Osaka University, Suita, Osaka 565-0871 Japan; Department of Material Science, Wakayama National College of Technology, 77 Noshima, Nada-cho, Gobo, Wakayama 644-0023 Japan; Osaka University Research Center for Ultra-high-voltage Electron Microscopy, Suita, Osaka 565-0871 Japan

**Keywords:** Biofilm, Extracellular polymeric substance, Ionic liquid, Scanning electron microscopy, *Streptococcus mutans*

## Abstract

Scanning electron microscopy (SEM) has been successfully used to image biofilms because of its high resolution and magnification. However, conventional SEM requires dehydration and metal coating of biological samples before observation, and because biofilms consist mainly of water, sample dehydration may influence the biofilm structure. When coated with an ionic liquid, which is a kind of salt that exists in the liquid state at room temperature, biological samples for SEM observation do not require dehydration or metal coating because ionic liquids do not evaporate under vacuum conditions and are electrically conductive. This study investigates the ability of ionic liquids to allow SEM observation of *Streptococcus mutans* biofilms compared with conventional coating methods. Two hydrophilic and two hydrophobic ionic liquids, all of which are electronic conductors, are used. Compared with samples prepared by the conventional method, the ionic-liquid-treated samples do not exhibit a fibrous extracellular matrix structure and cracking on the biofilm surface. The hydrophilic ionic liquids give clearer images of the biofilm structure than those of the hydrophobic ionic liquids. This study finds that ionic liquids are useful for allowing the observation of biofilms by SEM without preparation by dehydration and metal coating.

**Electronic supplementary material:**

The online version of this article (doi:10.1186/s13568-015-0097-4) contains supplementary material, which is available to authorized users.

## Introduction

Over 90% of bacteria in nature exist in robust, sessile communities known as biofilms (Vu et al. 2009), which can cause problems in a number of fields such as agriculture, industry, and medicine. Biofilm bacteria attach to surfaces and are surrounded by an extracellular matrix called the extracellular polymeric substance (EPS) (Costerton et al. 1999). Ninety percent of the dry weight mass of biofilms is composed of EPS, and the other major component of the biofilm matrix (up to 97%) is water (Sutherland 2001). The EPS has many functions, such as promoting the microbial adhesion and structural stability of the biofilm (Flemming et al. 2007; Stoodley et al. 2002), and enhancing biofilm resistance to antimicrobial agents. The EPS also protects against environmental stresses such as desiccation, oxidizing or charged biocides, and host immune defenses (Flemming and Wingender 2010). *Streptococcus mutans* is a Gram-positive, facultatively anaerobic bacterium, and an important etiologic agent of dental caries (Loesche 1986). *S. mutans* produces exopolysaccharide, which is synthesized by glucosyltransferases and the main constituent in the EPS of cariogenic biofilms (Bowen and Koo 2011; Hamada and Slade 1980). The EPS is essential for the initial adherence of *S. mutans* to tooth surfaces and facilitates the formation of a mature dental biofilm (Hamada and Slade 1980; Koo et al. 2003).

**Figure 1:**
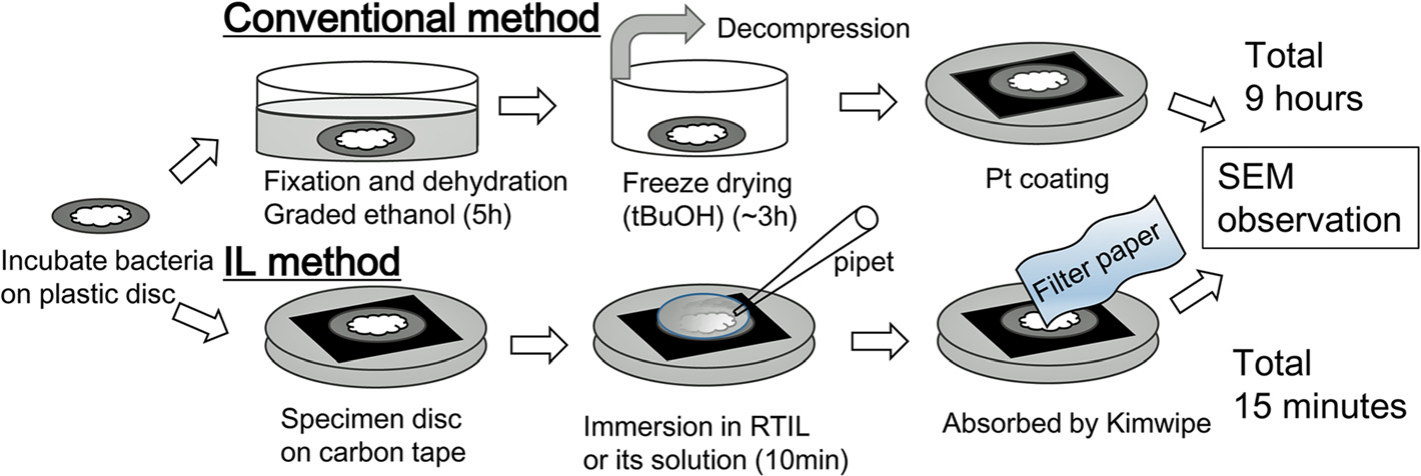
**Pretreatment of samples for SEM observation using the conventional and IL methods.**

Many early biofilm studies used scanning electron microscopy (SEM), which showed in detail the surface morphologies of microbial biofilms and their structure. Direct SEM observation clearly revealed that bacterial cells in a biofilm were extensively surrounded by fibrous or amorphous matrices, which represented the EPS (Marrie and Costerton 1984). However, when using a conventional scanning electron microscope, the sample chamber must be kept dry and under high vacuum during use, so the specimens are limited to nonvolatile, conductive materials (Tsuda et al. 2011b). If nonconducting samples are to be observed, such as biological specimens with a high water content, the samples must first be fixed with aldehyde, dehydrated with a graded alcohol series, dried, and then coated with a conductive film of a metal such as platinum prior to SEM observation (Figure 1). However, this dehydration process leads to considerable sample distortion and artifacts; for example, it causes the EPS to appear as fibers rather than as a thick gelatinous matrix surrounding the cells (Donlan and Costerton 2002). To avoid the draconian process of total sample dehydration required in conventional SEM, environmental SEM (ESEM), which can be used to visualize specimens in a wet or partially hydrated state, has been used to observe biofilm samples (Darkin et al. 2001; Priester et al. 2007). Other researchers have used variable-pressure SEM to observe biofilms (Weber et al. 2014).

Ionic liquids (ILs) are molten salts that remain in a liquid state even at room temperature (Torimoto et al. 2010). A large number of ILs have been synthesized and studied because of their many benefits such as negligible vapor pressure at room temperature and relatively high electronic conductivity (Tsuda et al. 2011a). It has been reported that ILs can behave as electronically conducting materials and enable SEM observation of samples without a metal or carbon coating (Kuwabata et al. 2006). Furthermore, replacing the water in samples with ILs allows the samples to remain wet during SEM observation because ILs resist evaporation even under vacuum conditions. The entire conventional biosample preparation process of dehydration, critical point drying and making the sample surface conductive can therefore be replaced by treatment with an IL. Such IL treatment has been demonstrated by the SEM observation of seaweed leaves swollen with water and subsequently soaked in ILs (Arimoto et al. 2008), as well as other biological samples such as fixed human culture cells (Ishigaki et al. 2011a), chromosomes (Dwiranti et al. 2012), and insects and pollen (Tsuda et al. 2011b). However, SEM observation of biofilm samples prepared by IL treatment has not yet been reported.

In this study, biofilms of *S. mutans* are treated with various kinds of ILs, and the conditions suitable for SEM observation of the IL-treated biofilms, including the optimum IL concentration and accelerating voltage used for SEM observations, are investigated.

## Materials and methods

### Bacterial strains and culture conditions

*Streptococcus mutans* MT8148 (JCM5175) was provided by Dr. Ooshima of Osaka University, Osaka, Japan. *S. mutans* MT8148 was grown in brain heart infusion (BHI) broth (Becton, Dickinson and Company, MD, USA) at 37°C under anaerobic conditions.

### Ionic liquids

**Figure 2:**
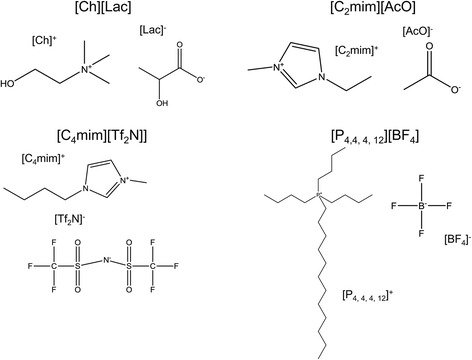
**Chemical structures of the ILs used in this study.**

The ILs used in this study were choline lactate ([Ch][Lac]), 1-ethyl-3-methylimidazolium acetate ([C_2_mim][AcO]), 1-butyl-3-methylimidazolium bis(trifluoromethanesulfonyl)amide ([C_4_mim][Tf_2_N]) and tri-*n*-butyldodecylphosphonium tetrafluoroborate ([P_4,4,4,12_][BF_4_]) (Figure 2). [Ch][Lac] and [C_2_mim][AcO] are hydrophilic ILs, while [C_4_mim][Tf_2_N] and [P_4,4,4,12_][BF_4_] are hydrophobic ILs (Table 1). [Ch][Lac] and [P_4,4,4,12_][BF_4_] were prepared according to the methods described in previous papers (Tsuda et al. 2011a; Tsuda et al. 2011b; Tsunashima and Sugiya 2007). The other two ILs, [C_2_mim][AcO] and [C_4_mim][Tf_2_N], were purchased from Kanto Chemical Co., Inc. (Tokyo, Japan). Prior to use, ILs were pretreated by vacuum drying at 80°C to remove impurities such as unreacted precursors and solvent.

**Table 1:** **Physicochemical properties of the ILs used in this study**

	**Hydrophilicity**	**Viscosity/cP**	**Formula weight**	**Reference**
[Ch][Lac]	+	895.0	193.24	Tsuda et al. 2014
[C_2_mim][AcO]	+	143.61	170.20	Tsuda et al. 2014
[C_4_mim][Tf_2_N]	-	50.5	419.37	McHale et al. 2008, Gardas and Coutinho 2008
[P_4,4,4,12_][BF_4_]	-	1310	458.45	Tsunashima and Sugiya 2007

### Biofilm formation

The medium used was BHI broth containing 0.5% sucrose. Biofilm formation was accomplished with a modified Robbins device as previously described (Noiri et al. 2003). Briefly, plastic disks were placed face down in the modified Robbins device after being coated with a 0.22 μm-filter-sterilized human saliva. Culture medium (400 ml) containing *S. mutans* cells (10^7^ cfu/ml) was perfused for 1 day using a peristaltic pump (SJ-1220; Atto Co., Tokyo, Japan) at a flow rate of 3.3 ml/min as described previously (Noiri et al. 2003). The culture medium was changed to fresh BHI medium without *S. mutans* and perfused for another 6 days. The medium was changed every 2 days.

### Conventional treatment to prepare samples for SEM observation

The specimens were immersed in half-strength Karnovsky’s solution (2% paraformaldehyde, 2.5% glutaraldehyde, pH 7.4) for 30 min. The fixed specimens were dehydrated using 50, 70, 80, 90, 95 and 100% (v/v) graded ethanol, and transferred into *t*-butyl alcohol. The specimens were then freeze-dried (JFD-320; JEOL, Tokyo, Japan). The samples were coated with platinum by sputtering with a plasma multicoater (PMC-5000; Meiwafosis, Tokyo, Japan) (Asahi et al. 2012). Samples that were treated with the conventional method were used as controls.

### Ionic liquid treatment of samples for SEM observation

Hydrophilic ILs were diluted with distilled water and hydrophobic ILs were diluted with ethanol prior to use. Samples were prepared without a dehydration process by dropping 50 μl of diluted ILs (1, 10, and 20% (v/v)) on top of each biofilm sample and leaving the sample for 10 min at room temperature. Any excess IL was then absorbed using Kimwipes (Figure 1). To evaluate the effect of fixation, some samples were fixed with 2.5% glutaraldehyde for 30 min prior to IL treatment.

### SEM observation

All specimens were examined with a scanning electron microscope (JSM-6390LV, JEOL, Tokyo, Japan) using the secondary electron emission mode with accelerating voltages of 1, 5, 10, and 20 kV. The magnifications used were × 1,500 and × 5,000.

### Transmission electron microscopy (TEM) observation

TEM observation was performed by slightly modifying the previously described sample preparation method (Asahi et al. 2014). Biofilm samples were treated with 10% hydrophilic ILs and left for 10 min at room temperature. Samples that were not treated with ILs were used as controls. The specimens were fixed first with 2.5% glutaraldehyde in 0.2 M cacodylate buffer containing 0.15% ruthenium red for 1 h, and then with 2% osmium tetroxide and 0.15% ruthenium red in 0.2 M cacodylate buffer. The fixed samples were dehydrated in a graded ethanol series and embedded in epoxy resin (Quetol 812 NissinEM, Tokyo, Japan). Ultrathin sections (70 nm) were then cut with a diamond knife (Nanotome Thick, Sakai Advanced Electron Microscope Research Center, Saitama, Japan) in an ultramicrotome (Ultrotome V, LKB, Stockholm, Sweden). These sections were mounted on copper grids (#100), stained with 2% uranyl acetate and 0.4% Sato’s lead stain, and examined using a transmission electron microscope (H800, Hitachi, Tokyo, Japan) with an accelerating voltage of 200 kV.

### Confocal laser scanning microscope (CLSM) observation

Biofilm samples were treated with 10% hydrophilic ILs and left for 10 min at room temperature. Samples that were not treated with ILs were used as controls. The specimens were washed and then stained with Concanavalin A-tetramethylrhodamine conjugate (Invitrogen, Carlsbad, CA, USA) and SYTO® 9 (Invitrogen) for 30 min in the dark. After rinsing in water, images were obtained using a CLSM (LSM700, Carl Zeiss, Munchen-Hallbergmoos, Germany). The scanning images were analyzed three-dimensionally using imaging software (Imaris®, Bitplane AG, Zurich, Switzerland). Fluorescent images were also quantified and exopolysaccharide levels were calculated as the ratio of the numbers of exopolysaccharides and cells. Significant differences between experimental and control groups were analyzed using Dunnett’s multiple comparison test (*P* < 0.01). Eight images per field per sample were acquired randomly. The experiment was independently repeated three times.

## Results

### Micromorphology of biofilms treated with platinum sputtering

**Figure 3:**
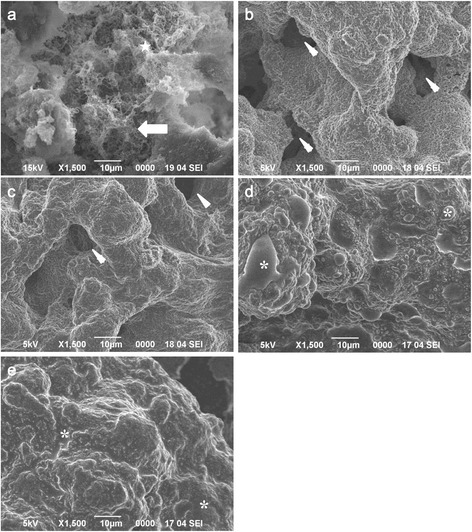
**SEM images of biofilms prepared for observation using hydrophilic and hydrophobic ILs. (a)**: SEM images of platinum-coated *S. mutans* biofilms. Fibrous extracellular matrix-like structures (star) were partially observed. In some areas of the biofilm surface, the cells were separated from the biofilm (arrow). Images of biofilms prepared for observation using hydrophilic ILs **(b)** [Ch][Lac] and **(c)** [C_2_mim][AcO], and hydrophobic ILs **(d)** [C_4_mim][Tf_2_N] and **(e)** [P_4,4,4,12_][BF_4_]. The concentration of all ILs was 10%. Some hydrophobic ILs were repelled by the biofilm surfaces and pooled on them. An asterisk indicates the accumulated IL. Arrowheads indicate the dark gaps thought to be water channels. Scale bars = 10 μm.

The SEM images of platinum-coated biofilms obtained in this study are similar to those of *S. mutans* biofilms reported by other researchers (Li et al. 2002; Pandit et al. 2011). The biofilms were composed of aggregated cocci, and fibriform extracellular matrix-like structures could be partially observed (Figure 3, Additional file 1: Figure S1). In some areas of the biofilm surface, the cells were almost separate from the biofilm (Figure 3a).

### SEM observation of biofilms treated with ILs

To investigate the effectiveness of fixing the biofilm with glutaraldehyde, an *S. mutans* biofilm was treated with glutaraldehyde and 10% [Ch][Lac]. Fixation with glutaraldehyde did not change the image quality compared with that of the control (Additional file 1: Figure S2), so subsequent samples were not fixed with glutaraldehyde.

The ability of the two hydrophilic and two hydrophobic ILs to image the *S. mutans* biofilm using SEM was compared by treating the biofilms with 10% ILs. Using the hydrophilic ILs, the quality of the SEM images was as high as those of the platinum-coated samples (Figure 3b and c), although the IL-treated biofilms exhibited a smooth surface that was quite different from the rough surface of the platinum-coated biofilms (Figure 3a). In addition, the fibriform extracellular matrix-like structures observed at the surface of the platinum-coated biofilms did not appear on the surface of the IL-treated biofilms. Cracks were barely visible in the IL-treated biofilms at magnifications of ×1500 and ×5000, and the dark gaps thought to be water channels were more discernible in the IL-treated biofilms than in the platinum-coated ones. In contrast, the SEM images of the samples prepared using hydrophobic ILs showed that these ILs were repelled by the biofilm surfaces and formed pools on them (Figure 3d and e).

**Figure 4:**
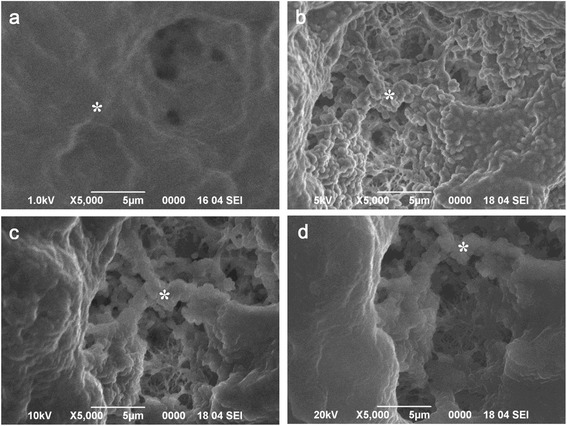
**SEM images recorded using various accelerating voltages on the same area of an IL-treated biofilm specimen.** The IL used was 1% [Ch][Lac]. The accelerating voltages were **(a)** 1 kV, **(b)** 5 kV, **(c)** 10 kV, and **(d)** 20 kV. At an accelerating voltage of 1 kV, it was difficult to focus the image. At 5 kV, the bacteria and biofilm outlines were observed clearly. Scale bars = 5 μm.

The images obtained were influenced by accelerating voltage (Figure 4). A lower accelerating voltage made it difficult to bring the image into focus, and limited the high-magnification observation of the IL-treated samples (Figure 4a). At a high voltage, however, the outlines of individual bacteria became obscured compared with those in the images obtained at lower voltage (Figure 4c and d). The optimum accelerating voltage for the IL-treated samples was around 5 kV (Figure 4b).

At some of the IL concentrations used in this study, the SEM images of the biofilm were clear (Additional file 1: Figure S3). However, for IL concentrations exceeding 20%, we observed that some hydrophilic IL accumulated on the surface of the biofilms (Additional file 1: Figure S3c).

### TEM observation of biofilms treated with ILs

**Figure 5:**
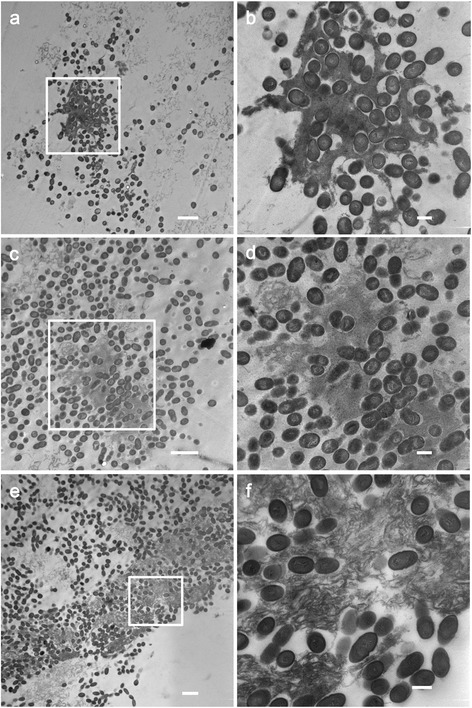
**TEM images of IL-treated** ***S. mutans*** **biofilms. (a)**, **(b)** control. Biofilms treated with **(c)**, **(d)** 10% [Ch][Lac] and **(e)**, **(f)** 10% [C_2_mim][AcO]. The EPS appears as electron-dense materials around the bacterial cell. **(a)**, **(c)**, **(e)** Low-magnification images, scale bars = 2 μm. **(b)**, **(d)**, **(f)** High-magnification images, scale bars = 500 nm.

To investigate the micromorphological effects of ILs on *S. mutans* biofilms, the samples were stained with ruthenium red because this polyanionic stain helps to maintain the structural integrity of biofilms during TEM observation (Barber et al. 1993). TEM analysis showed that the bacterial cells were surrounded by electron-dense fibrous matrices that were stained with ruthenium red (Figure 5). There was no appreciable difference in the images of the bacterial cell membrane, cell wall and cytoplasm obtained for the IL-treated biofilm when compared with those of the control biofilm (Additional file 1: Figure S4).

### CLSM observation of biofilms treated with ILs

**Figure 6:**
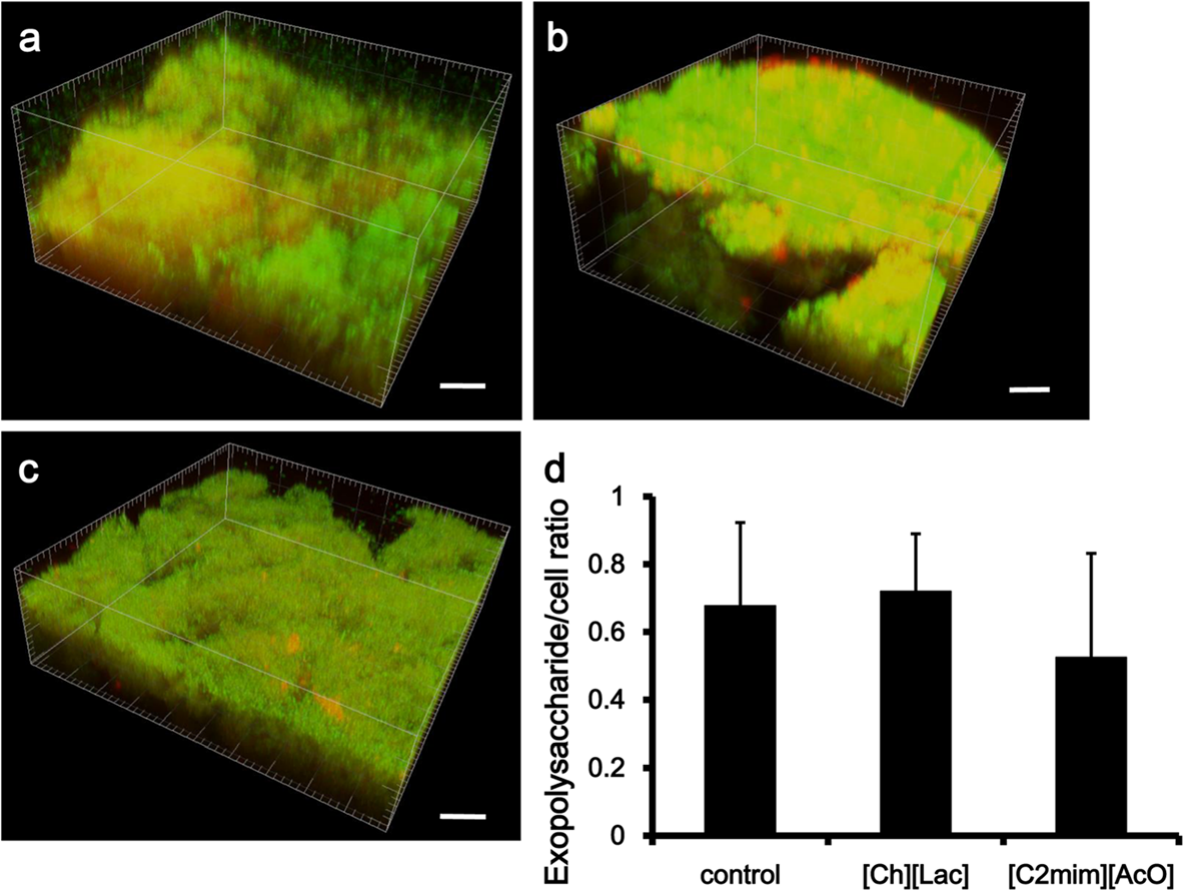
**CLSM observation of** ***S. mutans*** **biofilms treated with ILs.** Three-dimensional images of **(a)** control, **(b)** 10% [Ch][Lac]-treated and **(c)** 10% [C_2_mim][AcO]-treated biofilms. Scale bars = 10 μm. Exopolysaccharide is shown in red and bacterial cells in green. **(d)** Levels of exopolysaccharide represented by the ratio of the fluorescence of the exopolysaccharide to that of the cells. There was no significant difference between corresponding experimental and control groups (*P* > 0.01). Data are presented as the mean ± standard error (n = 8).

In all samples, the three-dimensional structure of the biofilms was examined and the EPS was found to be spatially heterogeneous. The effect of ILs on the EPS of *S. mutans* was evaluated, and no significant difference in the exopolysaccharide level was found between corresponding experimental and control groups (Figure 6).

## Discussion

To observe biological samples using SEM, wet specimens must be dehydrated before examination and coated by carbon or metal to provide electronic conductivity. The discovery that ILs can act as an electronically conducting material under vacuum conditions has led to the use of ILs to allow SEM observation of wet samples (Arimoto et al. 2008). In this study, we investigate the usefulness of the IL method for biofilm SEM observation. As shown by the results in the present study, clear SEM images of biofilms were obtained with a simple IL treatment that did not require a dehydration or metal deposition process. Some techniques like low-vacuum SEM and ESEM have been successfully used to observe wet samples, although problems have been reported when trying to obtain high-resolution ESEM images of biofilms. This is because of the lack of conductivity in the wet sample and because the focused electron beam appears to destroy the three-dimensional biofilm structure when the microscope is set to magnifications of ×10,000 and greater (Alhede et al. 2012). In this study, high-resolution images were obtained at ×10,000 magnification (Additional file 1: Figure S1) using biofilm samples prepared by the IL method.

The fibrous extracellular matrix structures observed on the biofilm surface of samples prepared by the conventional method were not detected on the surface of the IL-treated biofilms (Figure 3). It has been proposed that these fibrous structures are caused by the dehydration process in conventional sample preparation methods, so it appears that the biofilm structure observed after IL treatment has retained its form better than when the conventional method is used. Indeed, the SEM images of biofilms pretreated with ILs resemble those observed with low-vacuum SEM more closely than those obtained using samples prepared by the conventional technique (Darkin et al. 2001; Weber et al. 2014).

In the present study, we investigate the suitability of both hydrophilic and hydrophobic ILs for SEM observation of biofilms. Like other biological samples such as cultured human cells (Ishigaki et al. 2011b), hydrophilic ILs were found to be suitable for SEM observation of biofilms because of their affinity with the wet surface, but hydrophobic ILs were not (Figure 3). It is believed that the hydrophobic ILs are repelled by the water in the biofilm samples and are not able to replace it, causing the hydrophobic ILs to pool on the biofilm surface.

Higher concentrations of hydrophilic ILs also tended to pool on the biofilm surfaces (Additional file 1: Figure S3); this may arise from the higher viscosity of more concentrated solutions of ILs than that of more dilute ones (Table 1) (Gardas and Coutinho 2008; McHale et al. 2008). To prevent the ILs from accumulating on the surface of the biofilm, the ILs were therefore diluted. Using hydrous superabsorbent polymer particles for SEM observation after treatment with hydrophilic ILs, Tsuda et al. observed that more water in the particles was replaced with ILs upon using a higher concentration of ILs (Tsuda et al. 2014) and, when the substitution rate was higher, a decreased reduction of particle size was also observed. Therefore, a major factor to be considered when replacing the water in a biofilm is the IL concentration, and it is desirable to use a high concentration of ILs from the perspective of biofilm structure retention. Furthermore, considering that the major component of biofilms is water (Schmitt and Flemming 1999), the observation of an accumulation of ILs on the surface of the biofilm at high IL concentration may be the normal state of a biofilm that is covered with water (Additional file 1: Figure S3). It seems that the issue of efficiently replacing the water in the sample with ILs is an important one when using this method, so an IL that effectively replaces water is desired.

When using an IL as a conducting material for SEM observation, the accelerating voltage of the SEM is an important factor to consider (Ishigaki et al. 2011a). The images of the biofilms strongly depend on accelerating voltage (Figure 4), and it appears that the subsurface structure of the biofilm is observed better at higher accelerating voltages because the electrons are able to penetrate deeper into the specimen. This is an advantage of the IL method, and allows both the surface and subsurface structure to be imaged. When imaging IL-treated cultured human cells by SEM, the samples were easy to charge up at accelerating voltages of 10–15 kV, which are the optimum accelerating voltages for platinum-coated samples (Ishigaki et al. 2011a). In contrast, a high accelerating voltage did not charge up the biofilm samples treated with ILs in this study. To observe the outermost surface of the biofilm, 1 kV is believed to be the most suitable accelerating voltage, but such a low accelerating voltage gives poor image resolution.

Some ILs possess amphiphilic properties and behave as surfactants (Brown et al. 2013), especially surface-active ILs (SAILs) with long-chain alkyl groups (Galgano and El Seoud 2010). Rhamnolipid, a surfactant produced by *Pseudomonas aeruginosa*, is involved in the dispersion of biofilm cells by acting on the EPS (Boles et al. 2005). In this study, treatment with 10% IL for 10 min does not appear to disrupt the EPS within the biofilm (Figure 6). Among the ILs used in this study, [P_4,4,4,12_][BF_4_] is expected to behave as a SAIL because it has a long alkyl group. Conversely, [Ch][Lac] and [C_2_mim][AcO] both have short side chains and are thought to have no surface activity, so these ILs are unlikely to considerably alter the EPS.

It has been reported that some kinds of ILs have antimicrobial activity (Pham et al. 2010), which is affected by the length of the substituent; activity against cocci, rods, and fungi is lost when short substituent groups are present (Pernak et al. 2003). It has been suggested that the mechanism of the antimicrobial activity of ILs is the disruption of bacterial cell membranes (Venkata et al. 2012). However, destruction of the cell membranes in the biofilm bacteria treated with ILs was not observed by TEM in this study (Figure 5). Because [Ch][Lac] and [C_2_mim][AcO] possess short substituent groups and [Ch][Lac] contains carboxylate groups, it is believed that they will not act on the bacterial cell membrane. This is substantiated by the finding that [Ch][Lac] has a relatively high biocompatibility with fibroblast cells (Tsuda et al. 2011b).

Using ILs to prepare biofilm samples for SEM observation means that the biofilms do not require the extensive manipulation, fixation, dehydration, and coating that are used conventionally, and extreme sample dryness is also avoided. SEM observation using samples prepared with ILs does not require any special equipment and, importantly, high-resolution images can be obtained.

In conclusion, biofilm samples for SEM observation prepared by treatment with ILs can be readily visualized. Treatment with ILs can be added as an important sample preparation technique for the observation of biofilms by electron microscopy.

## Additional file

Additional file 1: Figure S1. High-magnification images of *S. mutans* biofilm using IL. (a) Control prepared for observation using the conventional method at × 10,000, (b) 1% [Ch][Lac], (c) 10% [C_2_mim][AcO] at × 10,000, and (d) 10% [Ch][Lac] at × 15, 000. Clear images were obtained at high magnification. Bars = 1 μm. Arrows indicate the fibriform extracellular matrix-like structures. **Figure S2.** SEM images of *S. mutans* biofilms prepared for observation using glutaraldehyde and IL. SEM images of *S. mutans* biofilms pretreated with (a) glutaraldehyde and 10% [Ch][Lac], and (b) 10% [Ch][Lac] (control). There were no differences detected between the SEM images of the glutaraldehyde-treated biofilm and control. Scale bars = 10 μm. **Figure S3.** SEM images of *S. mutans* biofilm using various concentrations of [C_2_mim][AcO]. (a) 1%, (b) 10%, and (c) 20%. The asterisk indicates the accumulated IL. Scale bars = 5 μm. **Figure S4.** High-magnification TEM images of *S. mutans* biofilms prepared for observation using (a) the conventional method (control), (b) 10% [Ch][Lac], and (c) 10% [C_2_mim][AcO]. Compared with those of the control biofilm, there was no difference incpf the images of the bacterial cell membrane, cell wall and cytoplasm for the IL-treated biofilms. Scale bars = 500 nm.
