# Supplementary material for: Simple observation of Streptococcus mutans biofilm by scanning electron microscopy using ionic liquids
Source: AMB Express. 2015 Jan 24;5:6. doi: 10.1186/s13568-015-0097-4 (PMC4305086; doi:10.1186/s13568-015-0097-4)
Supplement: Additional file 1: Figure S1. — High-magnification images of S. mutans biofilm using IL. (a) Control prepared for observation using the conventional method at × 10,000, (b) 1% [Ch][Lac], (c) 10% [C2mim][AcO] at × 10,000, and (d) 10% [Ch][Lac] at × 15, 000. Clear images were obtained at high magnification. Bars = 1 μm. Arrows indicate the fibriform extracellular matrix-like structures. Figure S2. SEM images of S. mutans biofilms prepared for observation using glutaraldehyde and IL. SEM images of S. mutans biofilms pretreated with (a) glutaraldehyde and 10% [Ch][Lac], and (b) 10% [Ch][Lac] (control). There were no differences detected between the SEM images of the glutaraldehyde-treated biofilm and control. Scale bars = 10 μm. Figure S3. SEM images of S. mutans biofilm using various concentrations of [C2mim][AcO]. (a) 1%, (b) 10%, and (c) 20%. The asterisk indicates the accumulated IL. Scale bars = 5 μm. Figure S4. High-magnification TEM images of S. mutans biofilms prepared for observation using (a) the conventional method (control), (b) 10% [Ch][Lac], and (c) 10% [C2mim][AcO]. Compared with those of the control biofilm, there was no difference incpf the images of the bacterial cell membrane, cell wall and cytoplasm for the IL-treated biofilms. Scale bars = 500 nm. [file 13568_2015_97_MOESM1_ESM.pdf]

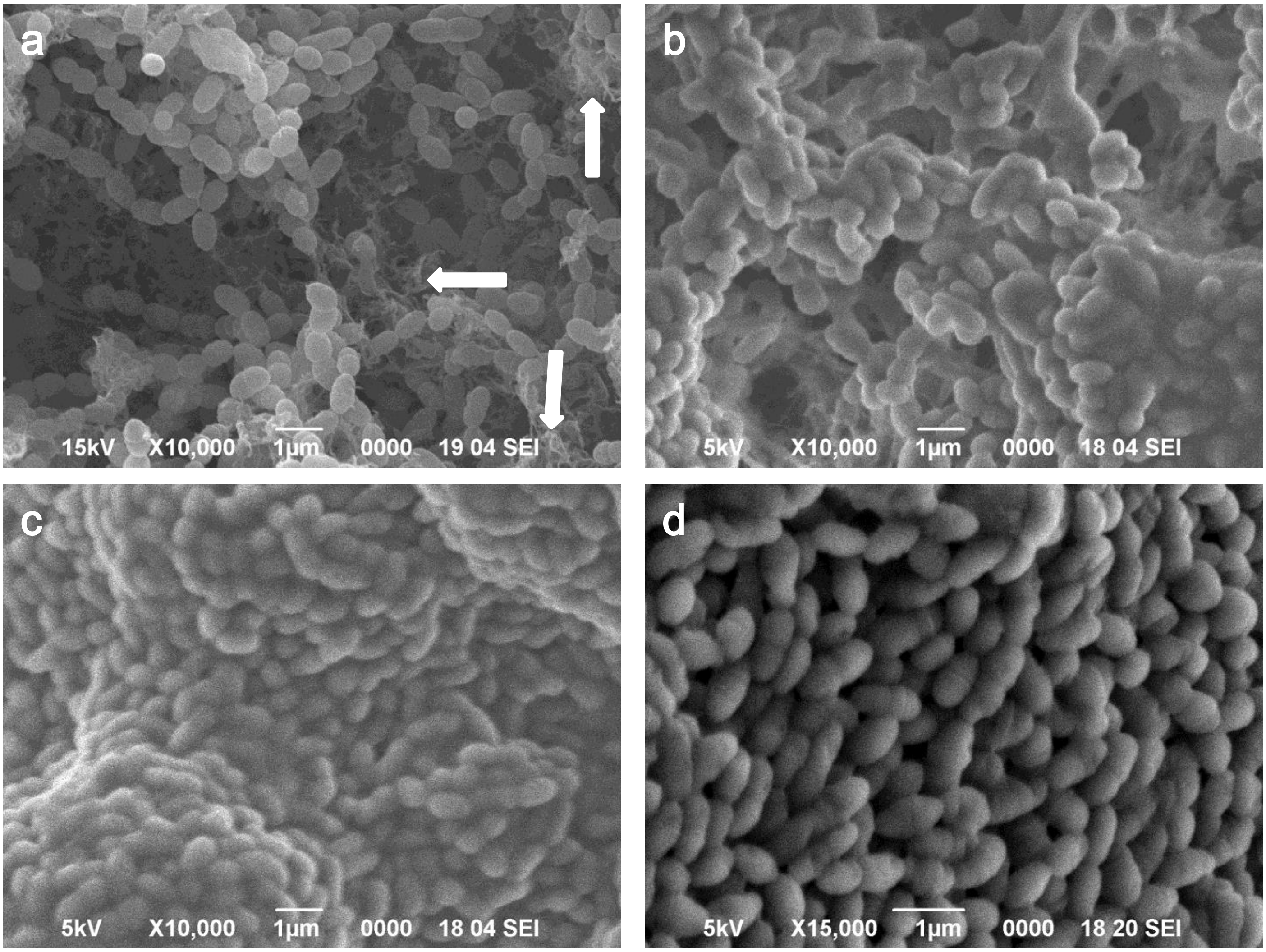

Figure S1 High-magnification images of *S. mutans* biofilm using IL.

(a): control using conventional method at ×10,000, (b): 1% [Ch][Lac], (c): 10% [C_2mim][AcO] at ×10,000 and (d): 10% [Ch][Lac] at ×15, 000. Clear images were obtained at high magnifications. Bars = 1 µm. Arrows indicate the fibriform extracellular matrix like structure.

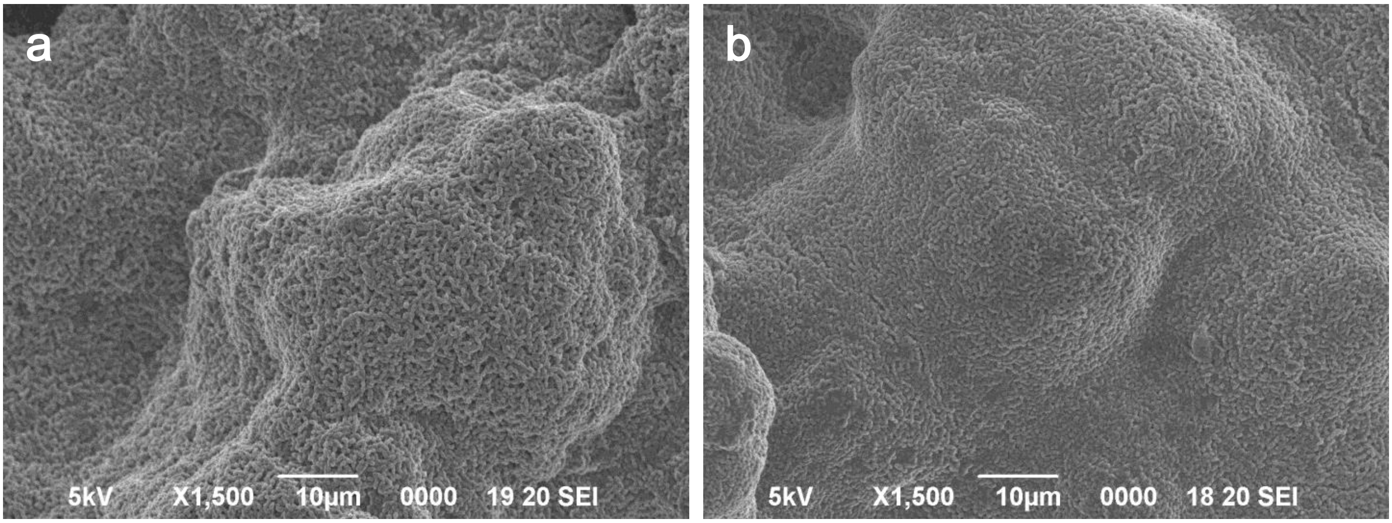

Figure S2 SEM images of *S. mutans* biofilms using glutaraldehyde and IL. SEM images of *S. mutans* biofilms pretreated with glutaraldehyde and 10% [Ch][Lac] (a) and with 10% [Ch][Lac] (control) (b). There were no differences on SEM images between glutaraldehyde-treated biofilm and control. Bars = 10 μm.

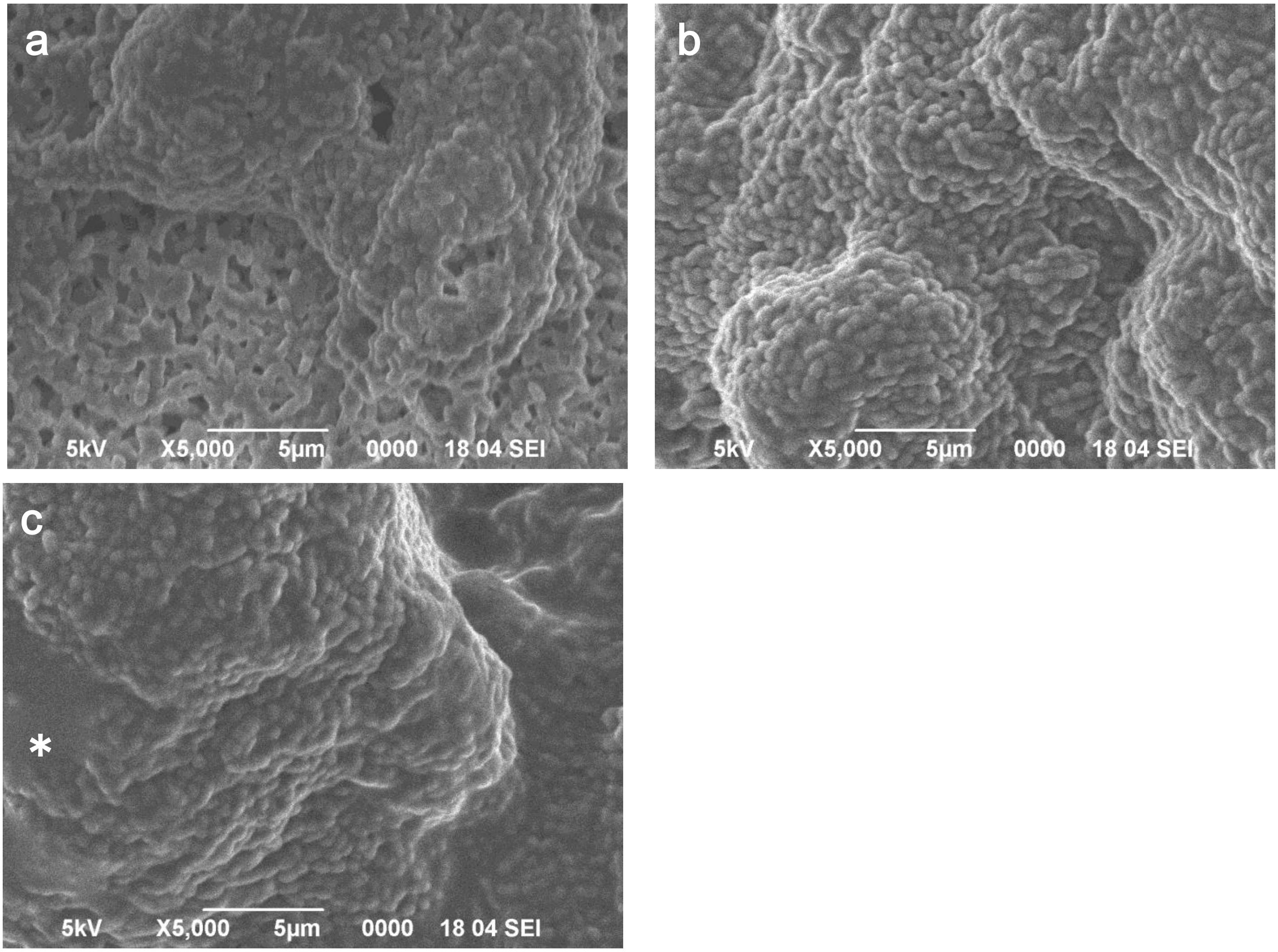

Figure S3 SEM images of *S. mutans* biofilm using IL of various concentrations. [C_2mim][AcO] was used at (a): 1%, (b): 10% and (c):20%. Asterisk indicates the accumulated IL. Bars = 5 µm.

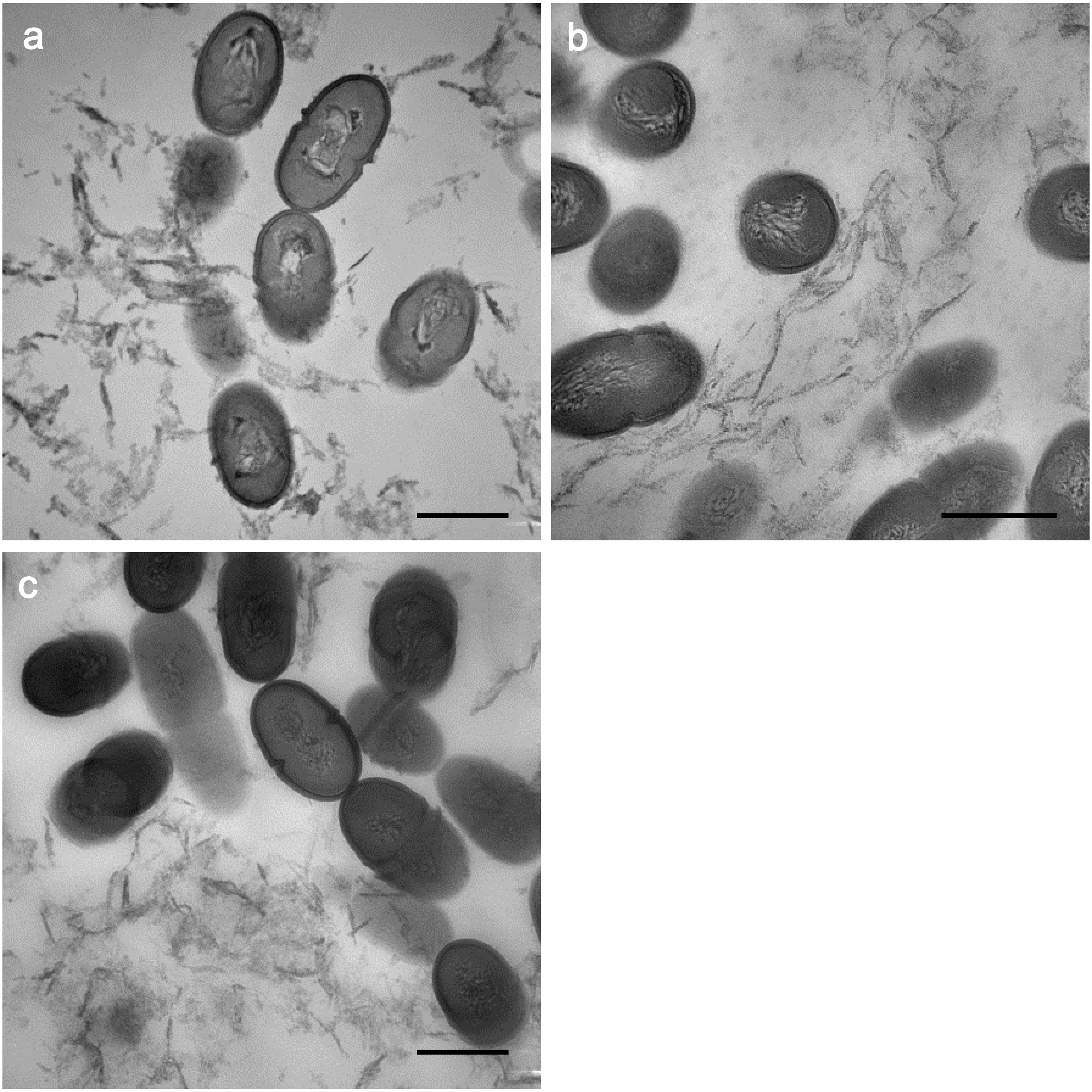

Figure S4 High-magnification images of *S. mutans* biofilm by TEM.

(a): control, (b): 10% [Ch][Lac] and (c): 10% [C_2mim][AcO]. Compared with the control biofilm, there was no difference in the images of the bacterial cell membrane, cell wall and cytoplasm for the IL-treated biofilm. Bars = 500 nm.
